# Supplementary material for: The influence of traceability label trust on consumers’ traceability pork purchasing behavior: Based on the moderating effect of food safety identification
Source: PLoS One. 2024 Jun 28;19(6):e0306041. doi: 10.1371/journal.pone.0306041 (PMC11213355; doi:10.1371/journal.pone.0306041)
Supplement: Attachment — Submitted filename: Response to Reviewers.docx [file pone.0306041.s002.docx]

Reviewer #1: Author have done a good job but only essential corrections needed before publications. I will be happy to revise it and check all these.
1. Introduction: There should be section wise representation of all headings included in a single para at the end.

Thanks for your advice. We have added this part in our manuscript. The content is as follows:

The subsequent structure of this paper is as follows: Section 2 covers the Literature Review, Section 3 describes the Methodology, Section 4 presents the Survey and Data, Section 5 deals with the Statistical Analysis, Section 6 discusses the Model Estimation Results and Analysis, Section 7 concludes with the Conclusions, and Section 8 offers Policy Recommendations.

2.LR: I unable to see the research gaps and objectives. Please include latest references which will give you an idea of work in different sectors. Few important papers needs to be added: DOI: 10.4018/978-1-4666-5202-6.ch022 ; https://doi.org/10.1504/IJBPSCM.2015.073770 ; Green supply chain management: A case of sugar industry in India. In: Proceedings of national conference on emerging challenges for sustainable business ; <https://doi.org/10.3390/w15081498> ; <https://doi.org/10.1504/IJLSM.2020.103862> ; <https://doi.org/10.1016/j.jclepro.2022.133311> ; <https://doi.org/10.1504/IJVCM.2017.086838> ; DOI: 10.4018/IJISSCM.287133 ; <https://doi.org/10.1007/s10479-021-04371-y> ; <https://doi.org/10.1007/s12063-022-00270-y> ; <https://doi.org/10.1177/09722629221107238> ; <https://doi.org/10.1108/IJPPM-02-2022-0080> ; <https://doi.org/10.1504/IJLEG.2023.130236> ; <https://doi.org/10.1504/IJMP.2018.090829> ; <https://doi.org/10.1007/s10668-022-02456-7> ; Supply chain performance of sugar industry using Regression analysis. International Journal of Engineering Technology Management and Applied Sciences.

Thanks for your advice and literature recommendations. We summarized them as follows:

There are barriers to e-applications in the Indian agri-food supply chain that are highly driven and lowly dependent [23]. In addition, questionnaires from 30 sugar mills in Uttar Pradesh and Uttarakhand, India, showed that the problems identified by the e-application are developing positive relationships and providing them with suggested IT solutions that will contribute to the overall development of farmers involved in sugarcane production [24]. There is also a study reviewing different individual and integrated approaches such as Multi-Criteria Decision Analysis (MCDA), multivariate, etc. to measure the problems at various stages of the supply chain of the olive oil industry in different countries [25]. Actually, Problems in the supply chain of other industries, such as the apparel industry export order fulfillment difficulties the most sensitive factor and in turn affects operating costs, changes in marketing strategies, changes in consumer purchasing patterns, and other factors, thus affecting profitability and employment cutoffs [26].

3.Methodology: Please check for proper citation of figures and tables in the text. In addition, there is no source given for figure and tables. If it is self then author should cite as “Source: Author Composition” or similar. Few of the latest papers should be cited in the text like: <https://doi.org/10.1007/s10479-023-05431-1> ; <https://doi.org/10.1111/opec.12287> ; <https://doi.org/10.3390/horticulturae8111018> ; <https://doi.org/10.1504/IJVCM.2022.122164> ; <https://doi.org/10.1177/097226292211432> ; <https://doi.org/10.3390/w15122274>

Thanks for your advice. We have cited “Source: Author Composition” for every figure and table.

4.Results and Discussion should be separate heading and must showcase the outputs and compare with other studies also.

Thank you for your suggestions. The results and discussion sections have been separated. And relevant literature has been added as follows:

With the increase in consumers' trust in traceability labels, the proportion of consumers who purchase traceable pork will increase. Similar results were found by Liang Fei et al. ([1]梁飞,马恒运,刘瑞峰.消费者信任对可追溯食品偏好和支付意愿影响研究——基于中国大中型城市可追溯富士苹果消费者的问卷调查[J].农业经济与管理,2019(06):85-98.). This is in line with the findings of Matzembacher’ s research that food traceability systems can increase the level of consumer utility and further enhance consumer trust in food quality (Matzembacher D E , Stangherlin I D C , Slongo L A ,et al.An integration of traceability elements and their impact in consumer's trust[J]. Food Control, 2018:S0956713518302391.DOI:10.1016/j.foodcont.2018.05.014.). In addition, the resident elderly in the composition of family members will have a significant negative impact on it, which is also largely in line with the findings of existing studies on traceable foods (应瑞瑶,侯博,陈秀娟等.消费者对可追溯食品信息属性的支付意愿分析:猪肉的案例[J].中国农村经济,2016(11):44-56.).

5.Implications should be added in this (Political, Practical and Social if any).

Thanks for your advice. We already have a policy recommendation section, which can be left out of the political and social implications.

6. Conclusion part is written in very short. Compare your study with other authors and write in conclusion and extend a bit more.

Thank you for your advice. We have added to the conclusions section.

7. Conclusions

Combined with a questionnaire survey on consumers' traceable pork purchase behavior in Shanghai, this study constructs a theoretical framework based on the influence of traceability label trust on consumers' traceable pork purchase behavior under the moderating effect of food safety identification. Through statistical analysis, quantitative analysis, etc. Empirical research, draws the following conclusions with certain robustness.

First, there is a certain correlation between traceability label trust and traceable pork purchase behavior. With the increase in consumers' trust in traceability labels, the proportion of consumers who purchase traceable pork will increase. Similar results were found by Liang Fei et al. [58]. Second, traceability label trust has a significant positive impact on consumers' traceable pork purchase behavior. Food safety identification can significantly strengthen, promote and other regulating effects on this influence process. The specific performance is that when consumers buy pork, the more attention they pay to the quality and safety of pork, the more they can strengthen the functions of traceability labels by providing information reference and determining after-the-fact responsibilities, building trust and further stimulating potential consumers to buy process of behavior. After changing the measurement method of the variable of food safety identification, that is, consumers are more assured of the quality and safety of the pork currently purchased, which also strengthens the positive impact of traceability label trust on consumers' traceable pork purchase behavior. This is in line with the findings of Matzembacher’ s research that food traceability systems can increase the level of consumer utility and further enhance consumer trust in food quality [15]. Third, the current consumers’ purchase behavior of traceable pork is significantly positively affected by consumption scenario preferences and price preferences. Consumers are more inclined to buy traceable pork in supermarkets or online platforms, and when the price level of household pork is lower, it is significantly less likely to purchase traceable pork. In addition, the resident elderly in the composition of family members will have a significant negative impact on it. In addition, the resident elderly in the composition of family members will have a significant negative impact on it, which is also largely in line with the findings of existing studies on traceable foods [59].

7. Limitations and future work should be included as a separate heading or may be a part of conclusion also in the last para.

In this study, the impact of traceability labeling trust on consumers' traceable pork purchasing behavior was verified through a questionnaire survey of 908 consumers in Shanghai. A Logit model was applied to analyze the impact of traceability labeling trust on consumers’ traceable pork purchasing behavior. Through existing studies, more studies have focused on the study of consumers' willingness to pay for traceable pork, and fewer studies have examined the impact of traceability labeling trust on consumers' traceable pork purchasing behavior, and this study also focuses on the time effect and regional effect, as well as the moderating effect of food safety identification, which is the innovation of this study. This study also focuses on the time and regional effects of this influence and the moderating effect of food safety identification, which is the innovation of this study. However, we also recognize that at this particular historical stage of the construction of China's pork traceability system, constrained by the fact that the level of the construction of China's pork traceability system is not yet high, in addition to investigating the effect of traceability label trust on consumers' traceable pork purchasing behaviors, we would like to validate the effect of traceability label querying behaviors and the content of the information of traceability label querying on consumers' continuous purchasing behaviors of traceable pork, which is also our next investigation and research plan.

8.Please make a proper structure of your manuscript like aligning with Introduction, LR, Gaps, Objectives, Methods and Materials, Analysis, Results and Discussion, Implications, Conclusion, Limitations and Future Scope and References.

Thank you for your advice. We have made systematic and comprehensive adjustments to the article.

Reviewer #2: Comments
Introduction
1.On page 3, lines 71-77, you are to give citations to the various studies listed.

Thanks for your advice. We have listed the citations to the various studies. As follows:

Poponi S, Arcese G, Mosconi EM, Pacchera F, Martucci O, Elmo GC. Multi-actor governance for a circular economy in the agri-food sector: bio-districts. Sustainability. 2021 Apr 23;13(9):4718.; Esposito B, Sessa MR, Sica D, Malandrino O. Towards circular economy in the agri-food sector. A systematic literature review. Sustainability. 2020 Sep 9;12(18):7401.; Stillitano T, Spada E, Iofrida N, Falcone G, De Luca AI. Sustainable agri-food processes and circular economy pathways in a life cycle perspective: State of the art of applicative research. Sustainability. 2021 Feb 25;13(5):2472.; Raheem D, Soltermann AT, Tamiozzo LV, Cogo A, Favén L, Punam NJ, Sarmiento CR, Rainosalo E, Picco F, Morla F, Nilson A. Partnership for international development: Finland–Argentina conference on circular economy and bioeconomy with emphasis on food sovereignty and sustainability. International Journal of Environmental Research and Public Health. 2022 Feb 4;19(3):1773.

2. From lines 117-140, the theoretical basis for this study is not strong and based mainly on comments and hypotheses by the authors and not literature. The authors should answer the question of what is the theory grounding this study. What theory is expected to be validated or otherwise at this study's end and how it fits well into its objectives?

Thanks for your advice. The theory grounding is as follows:

Based on trust theory, this study argues that consumers' trust in traceability labeling plays a crucial role in their purchasing decisions [46]. When consumers believe that traceability systems are reliable and effective in ensuring food safety, they are more likely to purchase traceable pork products This study examines how food safety identification moderates the effect of trust in traceability labeling on purchasing behavior. The study also draws on consumer behavior theory, which emphasizes the role of perceptions, attitudes, and decision-making processes in influencing how consumers respond to marketing stimuli [47]. Social cognitive theory suggests that consumer behavior is influenced by their observations of others, their sense of self-efficacy, and their perception of the consequences of their actions [48]. In the context of purchasing traceable pork, researchers examined how consumers' perceptions of others' purchasing behaviors and self-efficacy related to food safety influenced their own purchasing decisions.

3.The conceptual framework for the figure 1 should be relooked into. First, the food safety identification is to play a moderating role between traceability label trust and purchase behavior. However what is presented does not depict that. Again, the other variables included in the framework have not been explained in the text and hence need to be explained.

Thanks for your consideration.

The food safety labeling will play a moderating role between traceability labeling trust and purchasing behavior, as already indicated in Figure 1. The explanations for the other variables have been presented in Table 1.

Methodology
1.Explain why the logit model and not the probit model were used in the methodology. What makes Logit fit the probit model better for this study? Given the moderating effect, why didn’t you use Structural Equation modelling but Logit?

Thanks for your suggestions on the use of structural equation modeling. This study believes that the existing selected Logit model can meet the research needs. Specifically: this paper uses the 2017 and 2020 Shanghai consumer research data, which fails to do the tracking survey of the interviewed individuals, and there are some differences in the individual characteristics in the data, which belongs to the pseudo-panel data, which needs to be tested and selected regarding the form of model setting. Model (I) is a panel logit model with random effects, and model (II) is a mixed panel logit model. Based on the LR test results of model (I), the main model form should be selected as a mixed logit model. Models (III) and (IV) below actually add time control variables and area control variables to more control and verify the robustness of the model estimation results. Models (V), (VI), and (VII) then validate the moderating effect of food safety identification with or without considering time and region effects. Table 8 provides another more comprehensive test of model robustness. Combined with the changes in sign and significance level of the key explanatory variables, it can be seen that the results of the empirical analyses derived above for the impact of traceability labeling trust on consumers' traceability of pork, and the moderating effect of food safety identification are relatively robust. Based on this, this study did not choose structural equation modeling for the empirical research, but your suggestions will be fully absorbed. And we will try to use structural equation modeling to analyze the issue in-depth empirically in the follow-up study to see if we will find more valuable issues and results.

2.Under the study area, the authors should justify why the study area is the best-fit place for this study compared to other potential areas in China.

Thanks for your advice. We have justified the reason why we chose Shanghai as our study area.

As the economic center of China, Shanghai has a large pork consumption market and high consumer demand for pork quality and safety. Moreover, Shanghai's pork market has a strong industrial scale and influence, and its pig slaughtering, processing enterprises and sales links are relatively mature and representative. Therefore, the study of the construction of Shanghai pork traceability system can help to understand the safeguard measures of pork quality and safety in regions with higher economic development level, and can also provide reference for other cities and regions.

3.The sampling procedure here too is not clear. Was it convenient sampling? If yes, how did you get the people who have had pork purchase experience vis a vis ow many did you come across that had not pork purchase experience? Be clear on the sampling of respondents and the interviews.

In order to ensure the usability of the questionnaire data, this research was conducted in the form of face-to-face interviews in the field. The investigators were professors and students from the Shanghai Academy of Agricultural Sciences and the School of Economics and Management of Shanghai Ocean University. Moreover, we conducted special training and pre-survey of the questionnaire for the investigators before the formal survey. The respondents were all permanent residents of Shanghai and consumers who had purchased pork. Before the formal questionnaire survey, the respondents were asked whether they had purchased pork, and the questionnaire survey was conducted only when they received an affirmative answer. Respondents who completed the questionnaire were given a towel gift with a market price of about 10 yuan to mobilize the respondents to participate in the survey. At the same time, this also helps to ensure the quality of questionnaire completion. The survey locations were mainly chosen in supermarkets, farmers' markets and their neighborhoods. We considered the sample size of the questionnaire survey in each urban area according to the distribution of permanent residents in each urban area.

Results and Discussions
1.Table 2 is clumsy, and following through with the figures is challenging. The authors should redo it for clarity.

Thanks for your advice. We have made the following changes to Table 2 to make it clearer.

**Table 2** Basic characteristics of the samples

| **variable** | **Options** | **Ratio (%)** | **variable** | **Options** | **Ratio (%)** |
| --- | --- | --- | --- | --- | --- |
| gender | male | 49.78 | household registration | local | 51.21 |
|  | female | 50.22 |  | nonlocal | 48.79 |
| age | less than 30 years old | 39.65 | Family resident elders (60 years old and above): | Zero person | 73.57 |
|  | 30 to 39 years old | 29.30 |  | one person | 26.32 |
|  | 40 to 49 years old | 12.33 |  | Two person | 0.01 |
|  | 50 to 59 years old | 7.71 | Children in the family (under 15 years old): | Zero person | 62.89 |
|  | 60 years old and above | 11.01 |  | One person | 37.11 |
| education | Junior high school and below | 11.34 | Household Income in the Previous Year (After Taxes) | Below 100,000 yuan | 26.65 |
|  | Secondary School/High School | 12.67 |  | 10000 to 490000 yuan | 49.45 |
|  | college | 15.86 |  | More than 500,000 yuan | 23.90 |
|  | Undergraduate | 37.33 | Average monthly household purchases of pork | 0 to 8 catties | 54.30 |
|  | Graduate and above | 22.80 |  | 9 to 12 catties | 22.47 |
| Proportion of pork consumption in household livestock and poultry meat consumption | Below 10% | 17.18 |  | 13 to 16 catties | 13.22 |
|  | 10% to 29% | 30.95 |  | 17 to 20 catties | 4.96 |
|  | 30 to 49% | 26.76 |  | More than 20 catties | 5.07 |
|  | 50 to 69% | 16.30 | unit price of pork often purchased | Below 20 yuan | 33.70 |
|  | 70% and above | 8.81 |  | 20 to 30 yuan | 38.44 |
|  |  |  |  | more than 30 yuan | 27.86 |

2.Lines 270-272's argument is far-fetched. You just did cross-tabulation. For you to make that statement, undertake a chi-square test of the two cross-tabulated variables and also test the Cramers V to know the level of relationship in percentage terms.

Thanks for your advice. We have undertaken a chi-square test of the two cross-tabulated variables and also tested the Cramers V. The results as follows:

**Table 3** Cross-analysis of traceability label trust and consumer traceability pork purchase behavior

| **Traceability label** | **Traceable pork purchase** | | **No traceable pork was purchased** | |
| --- | --- | --- | --- | --- |
|  | Frequency | Proportion (%) | Frequency | Proportion (%) |
| trust | 408 | 48.46 | 434 | 51.54 |
| distrust | 12 | 18.18 | 54 | 81.82 |
| Total | 420 | 46.25 | 488 | 53.75 |
| Chi-square and Cramers V test | | | | |
|  | Value | df | P | |
| Pearson | 22.564 | 1 | 0.000 | |
| Cramers V | 0.158 | - | 0.000 | |

Source: Author Composition

3.The same should be done for Table 4.

Thanks for your advice. We have undertaken a chi-square test of the two cross-tabulated variables and also tested the Cramers V. The results as follows:

**Table 4** Cross analysis of food safety identification and traceability label trust

| **Food Safety Identification** | **Trust traceability label** | | **Distrust traceability label** | |
| --- | --- | --- | --- | --- |
|  | Frequency | Proportion (%) | Frequency | Proportion (%) |
| Concerned | 775 | 93.60 | 53 | 6.40 |
| Not concerned | 67 | 83.75 | 13 | 16.25 |
| Total | 842 | 92.73 | 66 | 7.27 |
| Chi-square and Cramers V test | | | | |
|  | Value | df | P |  |
| Pearson | 10.499 | 1 | 0.001 |  |
| Cramers V | 0.108 | - | 0.001 |  |

Source: Author Composition

4.The relevance of Table 6 and its difference from Table 5 is not explained. The authors should do well to explain.

Thanks for your advice.

The regression results related to Model 2, Model 3, and Model 4 in Table 5 and Table 6 are basically the same without too much difference. Therefore, it also explains that due to the small difference, the regression results in Table 6 and Table 5 are interpreted comprehensively.

5.The authors are unclear; the differences among models 1-5 and the moderating effect that was analyzed are unclear from the results of Table 7. 1 suggest structural equation modelling is used to moderate the effect to a more precise picture since the data is not a complete panel data.

In Table 5, model (I) is a panel logit model with random effects and model (II) is a mixed panel logit model. After adopting the mixed logit model, considering the completeness of the model construction, we cannot ignore the effects brought by the variables that do not change with individual changes. Therefore, time effect and area effect are introduced respectively to construct model (III) and model (IV) in Table 6. model (III) and model (IV) actually add time control variables and area control variables to control and verify the robustness of the model estimation results more. Model (V), model (VI), and model (VII), on the other hand, verify the moderating effect of food safety identification with or without considering the time and region effects, and the model estimation results are shown to be highly significant and credible.
Conclusions and Recommendations
1.Conclusions and recommendations could be better after the suggested corrections have been done.

Yes, thanks for your advice. The conclusions section has been modified to add a controlled validation with some of the studies, as well as to add some of the implications of the role of traceable food systems on society and practice.

Combined with a questionnaire survey on consumers' traceable pork purchase behavior in Shanghai, this study constructs a theoretical framework based on the influence of traceability label trust on consumers' traceable pork purchase behavior under the moderating effect of food safety identification. Through statistical analysis, quantitative analysis, etc. Empirical research, draws the following conclusions with certain robustness.

First, there is a certain correlation between traceability label trust and traceable pork purchase behavior. With the increase in consumers' trust in traceability labels, the proportion of consumers who purchase traceable pork will increase. Similar results were found by Liang Fei et al. [58]. Second, traceability label trust has a significant positive impact on consumers' traceable pork purchase behavior. Food safety identification can significantly strengthen, promote and other regulating effects on this influence process. The specific performance is that when consumers buy pork, the more attention they pay to the quality and safety of pork, the more they can strengthen the functions of traceability labels by providing information reference and determining after-the-fact responsibilities, building trust and further stimulating potential consumers to buy process of behavior. After changing the measurement method of the variable of food safety identification, that is, consumers are more assured of the quality and safety of the pork currently purchased, which also strengthens the positive impact of traceability label trust on consumers' traceable pork purchase behavior. This is in line with the findings of Matzembacher’ s research that food traceability systems can increase the level of consumer utility and further enhance consumer trust in food quality [15]. Third, the current consumers’ purchase behavior of traceable pork is significantly positively affected by consumption scenario preferences and price preferences. Consumers are more inclined to buy traceable pork in supermarkets or online platforms, and when the price level of household pork is lower, it is significantly less likely to purchase traceable pork. In addition, the resident elderly in the composition of family members will have a significant negative impact on it, which is also largely in line with the findings of existing studies on traceable foods [59].
